# Associations among Maturity, Accumulated Workload, Physiological, and Body Composition Factors in Youth Soccer Players: A Comparison between Playing Positions

**DOI:** 10.3390/biology11111605

**Published:** 2022-11-02

**Authors:** Hadi Nobari, Özgür Eken, Pablo Prieto-González, João Paulo Brito, Rafael Oliveira

**Affiliations:** 1 Department of Exercise Physiology, Faculty of Educational Sciences and Psychology, University of Mohaghegh Ardabili, Ardabil 56199-11367, Iran; 2 Department of Motor Performance, Faculty of Physical Education and Mountain Sports, Transilvania University of Braşov, 500068 Braşov, Romania; 3 Sepahan Football Club, Isfahan 81887-78473, Iran; 4 Faculty of Sport Sciences, University of Extremadura, 10003 Cáceres, Spain; 5 Department of Physical Education and Sport Teaching, Inonu University, 44000 Malatya, Turkey; 6 Health and Physical Education Department, Prince Sultan University, Riyadh 11586, Saudi Arabia; 7 Sports Science School of Rio Maior, Polytechnic Institute of Santarém, 2040-413 Rio Maior, Portugal; 8 Research Center in Sport Sciences, Health Sciences and Human Development, 5001-801 Vila Real, Portugal; 9 Life Quality Research Centre, 2040-413 Rio Maior, Portugal

**Keywords:** young, accumulated training load, football, maturity, peak height velocity, sprint

## Abstract

**Simple Summary:**

Considering the importance of the biological statuses of young soccer players, maturity offset could be essential for better physiological and body composition characterization of young athletes, and consequently, better load adjustment. Moreover, the knowledge about playing position differences and maturity statuses would increase the knowledge available to prescribe the proper intensity in training. Therefore, the present study examined the relationship among maturation variables such as peak height velocity, maturity offset, body composition, sprint ability, heart rate, and maximal oxygen consumption measures with variations in accumulated training loads in elite young soccer players. The main findings revealed that playing position did not influence any body composition measures, but there were differences in sprinting where central midfielders showed higher abilities. In addition, the maturity statuses and maximal oxygen consumptions were not positively associated with accumulated loads across the season. In opposition to previous research, the present study did not confirm the expected results, which suggests that the research could be influenced by the characteristics and environments of the soccer teams.

**Abstract:**

The purposes of this study were: (i) to analyze the correlation between accumulated workload (AW)—based on season periods—with maturity, linear sprints, maximum oxygen uptake (VO_2max_), maximum heart rate, and body composition; and (ii) to compare the playing positions based on the mentioned parameters. Twenty-one elite soccer players under the age of 14 participated in the study. They were divided into five groups based on playing positions. The in-season weekly AW was recorded for 26 weeks into two separated periods of 13 weeks (AW-1 and AW-2). Similarly, the following parameters were assessed: body mass, standing and sitting height, body mass index, body fat percentage, maturity offset, age at peak height velocity (PHV), sprinting ability (10 m and 30 m), and VO_2max_. The main significant differences between playing positions were found for weight, height, sitting height, and sprinting at 10 m and 30 m. No correlation was observed between AW (based on periods) and maturity or between VO_2max_ and AW-2. AW-1 denoted a large positive correlation with AW-2. AW-1 had a moderate negative correlation with VO_2max_, whereas PHV and maturity presented a strong negative correlation. Young soccer players’ maturity statuses and fitness levels do not imply differences between AW-1 and AW-2. However, the higher the AW in the first half of the season, the higher the AW in the second half. The absence of significant differences between player positions could be associated with the similar training regardless of the playing position. Moreover, soccer positively influences performance in short sprints (10 m), midfielders being the fastest.

## 1. Introduction

Soccer is a sport with several requirements to perform at the highest level, regardless of the type of competition (i.e., different categories and ages) and playing positions [1]. To attain the highest possible level, several measures must be considered, such as anthropometry, physical and physiological variables, soccer-specific skills [2,3], and in the case of young players, the peak height velocity and maturity.

Considering that young soccer players’ chronological and biological ages may not coincide, estimating the biological age is an essential aspect [4], and reinforces the use of anthropometric measures as well as fitness and physiological variables to acknowledge the status of the player [5]. Indeed, these variables have been reported as fundamental to a player’s performance [6].

Scientific evidence revealed that maturity status plays a significant role in identifying and selecting talent in youth soccer players [7,8,9]. Thus, as an essential indicator of biological development, maturity status should be integrated into the selection process of highly trained adolescent soccer players [10]. Furthermore, maturity status might also influence the physical fitness of young soccer players across the season [11]. After that, biological maturity is identified as the time required to reach the adult stage. During this stage, several sexual, morphological, neural, hormonal, somatic, and skeletal changes may occur [12,13]. The predicted maturity offset is defined as the age at which the highest increase in height occurs. This factor is known as age at peak height velocity (PHV) [13,14]. During the PHV stage, a larger variation in body height, ranging from 8.2 to 10.3 cm per year, was reported [15]. Research has indicated that the age at which PHV usually occurs on young male soccer players is between the age of 14 and 15 [16,17].

Fortunately, there are various ways to predict the maturity offset (PHV age), including measuring weight, standing height, sitting height, and leg length, and then performing a simple calculation [18]. However, it must be underlined that estimation errors of up to one year may occur in 95% of the cases with this method [18]. This detection may impact player status. For example, Goto et al. [19] found that more advanced maturing players had more opportunities to play in U9 and U10 teams, which was not verified in U11 and U14 teams.

In this regard, maximal oxygen consumption (VO_2max_) is a very important variable since it is helpful to distinguish soccer players’ levels from different age categories and playing positions [20]. Considering the characteristics of soccer, one of the most commonly used field tests is the 30–15 Intermittent Fitness Test (30–15 IFT) [21], which is progressive and performed until maximal volitional exhaustion [22]. In summary, this test consists of performing 30-s shuttle runs interspaced by 15 s of active recovery, where the speed increases in each shuttle run, the initial speed being 8 km/h [21].

Despite the importance of VO_2max_ for soccer players, the most relevant moments in a soccer match are related to high-intensity actions (e.g., sprint) [11,23]. Thus, other capacities, such as the sprinting ability, must be considered, but there is still little information on this subject [24]. Previous research highlighted the importance of developing technical skills, agility, and running in U13 and U14 categories [7]. Moreover, sprinting has been indicated as a talent identifier of young soccer players [25]. For instance, it was found that straight sprinting is the most frequent action used to score a goal for both attackers and assists among young soccer players [26].

Despite existing evidence about maturity, biological, and physical fitness variables, the information regarding the relationship between the mentioned aspects and load variations through the season is limited [27]. For instance, one study analyzed the relationship between cardiorespiratory fitness and the accumulated training load parameters over a four-month in-season period in U10 soccer players, and showed that accumulated load was strongly associated with changes in aerobic power [28]. Another study conducted with different age categories (U12, U13, U14, and U15) found that the accumulated training, maturity, and initial physical fitness status explained only small and inconsistent proportions of the observed physical fitness modifications after a full season [29]. Similarly, a study conducted with different age categories (U14, U15, U16, U17) showed that maturation status has a moderate effect on match load [30]. Interestingly, a recent study on U16 soccer players showed that physical fitness changes after a full season seemed to be influenced by both accumulated training intensity and maturation status [11]. This study highlighted that accumulated training load and maturation status play a critical role on physical fitness improvements through the season [11]. This finding is very relevant since sprinting is considered to be an ability that improves with age [31].

Furthermore, knowing the evolution through the sports season of the variables mentioned above is crucial since it can influence the training process and sports performance during the competition. Therefore, all variables must be monitored and compared frequently during the different phases of the season. Unfortunately, research into this topic seems inconclusive regarding establishing differences based on training loads, anthropometry, and physical and physiological aspects according to players’ positions in young soccer players [1,2,5,11]. Moreover, most of the literature splits the different mentioned variables and does not use them simultaneously [5].

Therefore, the purposes of this study were twofold: (i) to perform a correlation analysis between accumulated workload (AW) based on periods (i.e., first and second halves of the season) and maturity (i.e., maturity offset and PHV), linear sprints (i.e., 10 m and 30 m), VO_2max_, maximum heart rate (HR_max_) and body composition variables; and (ii) to compare playing positions based on the previously mentioned parameters.

Since previous research conducted on U16 soccer players—that analyzed the relationships between maturity status and training load with variations in physiological variables—showed that aerobic and speed performance improved over the season and had a strong association with PHV, as well as an increased accumulated load to the end of the season [5,32], it was hypothesized that changes in accumulated training load through the season will occur and that both accumulated training load and maturation may partially explain variations in physical fitness variables during the competition in young soccer players.

## 2. Materials and Methods

### 2.1. Participants

Twenty-one male elite soccer players under the age of 14 (mean ± standard deviation (SD); age: 13.26 ± 0.20 years; height: 165.80 ± 11.67 cm; body mass: 50.70  ±  7.56 kg; VO_2max_, 44.23  ±  2.80 mL.kg^−1^ min^−1^) participated in the study. These players competed in the best premier league of Iran. In this study, players were divided into five groups based on game positions. The participants were four central defenders (CD), four central midfielders (CM), four wide defenders (WD), five wide midfielders (WM), and three strikers (ST). The inclusion criteria for this study were: each player’s information was reported for at least 90 percent of the training sessions; players were not allowed to participate in any training other than team training; and players who did not attend every week’s match were considered to have balanced training with other players. Before beginning the research, permission was received from the parents and athletes, as well as from the ethical committee of the University of Mohaghegh Ardabili. This research was conducted in accordance with the Declaration of Helsinki Principles.

Through the G*Power software (University of Düsseldorf, Düsseldorf, Germany) [33], a post hoc correlation (correlation: Point biserial model) analysis was used to calculate sample size power with the following information: α err prob = 0.05, power (1-β err prob) = 0.80, and effect size = 0.5. This effect size is considered due to previous studies reporting large to very large correlations between physiological variables in soccer players [32,34,35]. Finally, the results revealed that actual power was 81.7 % with a sample size of 21 players.

### 2.2. Experimental Approach to the Problem

This study was performed as a quasi-experimental and cohort research conducted on a cross-sectional basis. The researchers closely watched the players during the entire season, and then, following the completion of the season in which the competitions were held, assessments were carried out. The ongoing research was carried on for a total of 26 weeks. Weekly training was at least three sessions per week with one match. Most of the training and matches were accomplished in the afternoon. The entire season was split into two halves: first half of the season (July to October, weeks 1 to 13) and second half of the season (October to January, weeks 14 to 26). Daily training load data were analyzed to report changes in weekly acute workload (AW) during the first and second halves of the season. Throughout the entirety of each test period, players had their measurements collected on several consecutive days. On the first day of the test, each participant’s anthropometric and body composition characteristics were calculated. This series of measurements was conducted first thing in the morning [34].

### 2.3. Data Measurement and Variables

#### 2.3.1. Anthropometric Measurements

Three variables were evaluated to assess the anthropometric measures: weight, standing height, and sitting height. Height (cm) was measured using a stadiometer (Type SECA 225, Hamburg, Germany) to the nearest 0.1 cm. Body mass index (BMI) was calculated by dividing body weight (kg) by height squared (m). The measurements were performed in accordance with the recommendations of the International Society for the Advancement of Kinanthropometry (ISAK) before breakfast [36,37]. The measurement of seat height has previously been documented in detail [34,38]. Subjects stood barefoot and in light clothing for height, sitting height, and weight measurements.

Based on the information collected above and using the Mirwald formula [18], the maturity offset and age at PHV were determined [18]: maturity offset = −9.236 + 0.0002708 (leg length × sitting height) − 0.001663 (age × leg length) + 0.007216 (age × sitting height) + 0.02292 (weight by height ratio), where R = 0.94, R2 = 0.891, and SEE = 0.592) and for leg length = standing height (cm) − sitting height (cm) was used.

To measure body fat percentage, triceps, and subscapular fat thickness, the Jackson and Pollock method was used [18,39]. Data were collected by a skinfold caliper Lafayette Instrument Company (Lafayette, IN, USA) with an accuracy of 0.1 mm. All measurements were performed by the same researcher on the right side of the body. Based on the study conducted by Nobari et al. (2020), the technical measurement error was also considered [38].

#### 2.3.2. Training Load Calculation

Each athlete was questioned individually, half an hour after each training session on the Category-Ratio-10 Borg scale, about the feeling of the training intensity. On this scale, 1 represents a very easy training session, and 10 represents a highly intense training session [40]. For better clarity, players were previously familiarized with the scale during two years at the club. Then, workload (WL) was determined for each training session, taking the rated perceived exertion (RPE) multiplied by session duration to generate the session-RPE (s-RPE). These data were utilized to obtain AW per week [40,41] for the first (AW1) and second (AW2) halves of the season. AW1 and AW2 were calculated by summing all weeks from the first and second halves of the season, respectively.

#### 2.3.3. Sprint Test

For the sprint test, a digital timer connected to two photocells was mounted at hip level. Players warmed up for around 10 min, consisting of a combination of running exercises (4–5 min at light intensity), followed by 4–5 min of dynamic mobility emphasizing the lower-extremity muscle groups (gastrocnemius, quadriceps, hip flexors, adductors, hamstrings, and gluteals) and then sprinting for 5 to 10 m. Players warmed up for around 10 min, first running slowly, then ABC movement, and then sprinting for 5 to 10 m. After the warmup, participants were positioned 70 cm in front of the starting line. To determine the sprint time, the test was conducted at a distance of 30 m [42]. The participants’ 10 m and 30 m times were determined. The best value from three trials was utilized for statistical analysis. The rest between trials was three minutes. The coach monitored all phases of the examination. Sprint tests were conducted utilizing the Newtest Powertimer 300 series test equipment (Tyrnava, Finland) for this investigation. In this study, the ICC was equivalent to two 0.87 replicates.

#### 2.3.4. Aerobic Fitness Status and Heat Rate Measurements

The 30–15 Interval Fitness Test (30–15 IFT) was used to assess athletes’ aerobic fitness, applying the original 40-m version of the 30–15 IFT. This test requires athletes to run for 30 s and then recover (i.e., walk) for 15 s. The initial speed was 8 km/h, and it increased by 0.5 km/h per level. The test finished when the participant failed to reach the 3-m field three times or after volitional exhaustion [43,44]. The 30–15 IFT was used to estimate the VO_2max_ with the following formula: VO_2max_ (mL.kg^−1^.min^−1^) = 28.3 − (2.15 × 1) − (0.741 × 14 yrs) − (0.0357 × Weight) + (0.0586 × 14 yrs × VIFT) + (1.03 × VIFT), where VIFT= is the final running speed (km/h) [43].

The maximum heart rate (HR_max_) variable was measured with a Xiaomi Mi-Band 3 (Xiaomi, Beijing, China) while each player was performing the 30–15 IFT. The highest value obtained in the final stage of the test was used for analysis.

### 2.4. Statistical Analysis

Statistical analysis was performed using GraphPad Prism 8.0.1 (GraphPad Software Inc., San Diego, CA, USA). The significance level was set at *p* ≤ 0.05. Shapiro–Wilk was used to assess the normality. Since the data were not normally distributed, the variables were summarized as mean ± standard deviation (SD). Therefore, nonparametric tests were conducted. Kruskal-Wallis testing was conducted to verify the existence of significant group differences between groups. To address pairwise comparisons in variables, a Mann-Whitney U test was used. The Hopkins threshold was used to quantify the effect size (ES) as follows: <0.2 = trivial, 0.2 to 0.6 = small, >0.6 to 1.2 = medium, >1.2 to 2.0 = large, >2.0 to 4.0 = very large, and >4.0, almost perfect [45]. Spearman correlation analysis was performed between training load parameters (AW-1 and AW-2) periods using PHV, maturity, 10 m, 30 m, and VO_2max_ factors. The correlation coefficient was interpreted as follows: <0.1 = trivial; 0.1–0.3 = small; >0.3–0.5 = moderate; >0.5–0.7 = large; >0.7–0.9 = very large; and >0.9 = nearly perfect [46]. The significance level was considered as *p* ≤ 0.05.

## 3. Results

Descriptive characteristics of the elite youth soccer players are shown in Table 1, and pairwise comparisons between the subgroups (central defenders vs. central midfielder vs. striker vs. wide defender vs. wide midfielder) based on age, anthropometric and maturational characteristics, and results obtained in the fitness tests performed are shown in Table 2.

As for the comparisons (see Table 2), statistically significant differences between playing positions were found for weight (*p* = 0.032; ES = 0.43), height (*p* = 0.016; ES = 2.16), sit height (*p* = 0.039; ES = 1.64), and sprint at 10 m (*p* = 0.025; ES = 0.48) and 30 m (*p* = 0.017; ES = 0.54) (see Table 2).

In contrast, no significant differences between playing position were found for the following variables: age (*p* = 0.885; ES = 0.96), PHV (*p* = 0.763; ES = 0.81), maturity (*p* = 0.898; ES = 0.98), weight/height ratio (*p* = 0.315; ES = 0.45), height/weight ratio (*p* = 0.449; ES = 0.29), triceps (*p* = 0.688; ES = 0.72), subscapular (*p* = 0.178; ES = 0.84), BF % (*p* = 0.385; ES = 0.20), AW- first half of season (*p* = 0.210; ES = 1.90), AW- second half of season (*p* = 0.904; ES = 0.99), V IFT (*p* = 0.760; ES = 0.81), VO_2max_ (*p* = 0.700; ES = 0.74), and HR_max_ (*p* = 0.959; ES = 1.07) (see Table 2).

The correlation analysis between training loads parameters (AW) based on periods (first and second halves of the season) with maturity (maturity offset and PHV), 10 m, 30 m, and VO_2max_ variable is shown in Table 3. The results were: PHV was negatively and very largely correlated to maturity (r = −0.866 very large, CI 95% {−0.59 to 0.28}; *p* ≤ 0.0001). It was revealed that 10 m was positively and very largely correlated to 30 m (r = 0.769 very large, CI 95% {0.48 to 0.90}; *p* ≤ 0.0001). AW-1 was negatively and moderately correlated to VO_2max_ (r = −0.480 moderate, CI 95% {−0.76 to −0.03}; *p* = 0.03). AW-1 was positively and largely correlated to AW-2 (r = 0.551 large, CI 95% {0.13 to 0.80}; *p*= 0.03). HR_max_ was negatively and moderately correlated to BF% (r = −0.469 moderate, CI 95% {−0.76 to −0.01}; *p* = 0.03). VO_2max_ was positively and moderately correlated to HR_max_ (r = 0.490 moderate, CI 95% {0.07 to 0.77}; *p* = 0.02). TD-average was positively and nearly perfectly related to the total training duration (TD-top) (r = 1.000 nearly perfect, CI 95% {1.00 to 1.00}; *p* ≤ 0.0001). It was determined that s-RPE was positively and very large related to RPE (r = 0.853 very large, CI 95% {0.65 to 0.94}; *p* ≤ 0.0001). s-RPE was positively and largely related to TD-top (r = 0.666 large, CI 95% {0.30 to 0.86}; *p* = 0.000). Moreover, s-RPE was positively and largely related to average training duration (TD-average) (r = 0.666 large, CI 95% {0.30 to 0.86}; *p* = 0.000).

## 4. Discussion

The purposes of this study were twofold: (i) to perform a correlation analysis between accumulated workload (AW) based on periods (i.e., first and second halves of the season) with maturity (i.e., maturity offset and PHV), linear sprints (i.e., 10 m and 30 m), VO_2max_, maximum heart rate (HR_max_), and body composition variables; and (ii) to compare all variables based on player position.

Regarding the first aim of this study, it was found that there was no correlation between accumulated workload (AW) based on periods (i.e., first and second halves of the season) with maturity (i.e., maturity offset and PHV) and linear sprints (i.e., 10 m and 30 m). In agreement with other studies [5,47], it could be hypothesized that the magnitude of maturity status and physical fitness level of performance would dictate the relationship between the accumulated workload, the maturity, and the sprint velocity of U14 team. However, AW-1 was positive and largely correlated to AW-2, which means the greater the AW in the first half of the season, the greater the AW in the second half. It seems that being exposed to a higher AW in the first half of the season may lead to greater physical and functional development for players, allowing them to have higher loads in the second half of the season. However, this is not supported by the negative and moderate correlation found between AW-1 and VO_2max_ and the absence of a relationship between VO_2max_ and AW-2. This inverse relationship between the AW-1 and VO_2max_ can eventually be interpreted by the fatigue accumulated in the first half of the season [48]. Although there are no significant differences, the AW absolute values in the first half were higher than in the second. The strong and negative correlation found between PHV and maturity is in agreement with the expected growth coincidental with the onset of puberty. However, it may happen in different momentums and at different rates [49]. The highest record of this rapid growth phenomenon is also known as the peak height velocity (usually at ~14 years old for males), which corroborates the almost coincident results found in the present study for the ages of PHV and maturity [50].

The other relationships found between performance at 10 m and 30 m indicate the athletes’ ability to maintain performance in both distances. Specifically, the type of training that positively affected sprint performance in the 10-m distance also seems to have an effect on the 30-m performance [51,52]. It is known that the performance of the initial acceleration (0–10 m) is affected mainly by concentric action and power performance [53]. In contrast, the maximal velocity phase is also affected by muscle-tendon stiffness [54]. Therefore, it seems that the initial acceleration was maintained at 30 m.

HR_max_ was negatively and moderately correlated to BF%. This suggests that youth soccer players with higher BF% reach lower HR_max_ values. However, although the HR_max_ was positively and moderately correlated to VO_2max_, which was also evident in previous research [55], there was no relationship between the cardiorespiratory capacity and the BF%. Body composition may have considerable implications on aerobic and anaerobic performance [56]. Therefore, the determination of predicted VO_2max_ normalized to body mass and not to BF% may have conditioned this relationship [57,58].

Despite these results, it has been documented that the maturity level of youth soccer players is associated with better physical performance [59,60]. With the achievement of the PHV, the players tend to present a greater development of their aerobic capacity. However, this development may present a delay in relation to the PHV, that is, it may occur somewhat later, along with the development of maximal aerobic power, strength, and power [32,61].

Players of this study were categorized into five playing positions common in soccer and used in previous studies [5,47]: central defenders, central midfielders, wide defenders, wide midfielders, and strikers. As reported in several studies, anthropometric variables, such as body mass, height, and sitting height, were statistically significant between playing positions [47,62,63]. This result may be due to differences in the maturational development of young players of peripubertal age [64]. The differences found in the anthropometric variables between playing positions may also be a result of the specificity and requirements of playing positions, which are already noticeable at these ages [52,61,63]. CD and strikers were the heaviest compared to WM. Moreover, WM and CM were the shortest among the remaining positions. Height influences the performance of soccer players, especially on reaching high balls. Thus, a lower height can be a disadvantage to players in the current study when playing high balls. In the weight/height ratio, only the CD differs from the CM with a very large effect size, although the same result is not verified in the inverse ratio. In other words, the CMs have a lower height than the CDs, which may result from the specific playing position. These results may show a deliberate tendency of the team coach to prefer tall and heavy players for defense and goalkeeping positions.

Despite the differences found in the anthropometric variables of weight, height, and sit height between playing positions, there were no differences in PHV, maturity, weight/height, or height/weight ratios. We consider that the magnitude of these differences did not influence the PHV, maturity, weight/height, or height/weight ratios between playing positions. However, our sample is composed of subjects at mid-puberty (U-14), and it could be expected to find differences between early and late puberty among individuals who play in different field positions [19]. Given this, more studies should be conducted to ensure greater information about weight, height, and sit height influencing PHV and maturity.

When analyzing the body composition variables (e.g., triceps, subscapularis, and BF %), differences were only found between the subscapularis skinfold CD vs. WM. The absence of a significant difference between the players’ positions could be because all players receive similar training, and there is still no difference in the specific requirements of each field position. Masocha and Katanha [65] also reported a similar result.

Comparing the performance in the 10-m and 30-m linear sprints, between player positions, differences were found between the CD vs. CM and CD vs. WM with very large effect size, and between CDs vs. WD only in the 10-m. The results showed a significant influence of playing positions on linear-running sprint performance. Midfielders reached significantly higher performance levels, which agree with other studies in peripubertal youth [5,32,65,66]. The possible explanation of this result could be associated with the demands of the positional play characteristics at these ages.

Despite the differences found between player positions, some findings are difficult to interpret. Considering the anthropometry, maturation, body composition, and physical performance assessment of the soccer players is not possible to identify a clear pattern for each player’s position. In this context, the present study contributes to the existing literature, providing information about the variables mentioned in youth athletes of one professional soccer club.

The present study contains some limitations. First, the small sample size of only one Iranian team implies that the results cannot be generalized. Moreover, previous studies highlighted that playing time [67] or playing status (starters versus nonstarters) [38] could have an impact on training load. However, the present study did not address those factors. Furthermore, training load parameters were only assessed by RPE, while other running and accelerometry-based variables could have strengthened the present research, as pointed out in a previous study [2].

Despite the previous limitations, the strengths of this study are related to the limited number of studies on U14 soccer players and the fact that playing positions presented differences in anthropometric and sprint variables.

## 5. Conclusions

From the study results, it is concluded that maturity status and physical fitness level did not promote differences between the AW-1 and the AW-2. However, the greater the AW in the first half of the season, the greater the AW in the second half of the season.

No differences in PHV, maturity, weight/height, or height/weight ratios were found.

The absence of a significant difference in body composition variables between the players’ positions (except for CD vs. WM in subscapularis skinfold) could means that all players receive similar training and that there is still no significant difference in the specific requirements of each field position.

Since it could be expected to find differences between early and late maturing players of different field positions, we consider that in the present study, these differences did not reach sufficient magnitude to produce significant differences between groups of players based on the positions they play due to the small sample size.

For the performance in the 10-m and 30-m linear sprints, the playing positions showed a significant influence on linear-running sprint performance. Midfielders reached significantly higher performance levels.

The results of the present study highlight the effects of training load and physical changes over the season on performance, which should be considered by practitioners and researchers in their future work.

## Figures and Tables

**Table 1:** Descriptive characteristics of the subjects.

Variables	Mean ± SD
Height (cm)	165.8 ± 11.6
Body mass (kg)	50.7 ± 7.5
Age (years)	13.2 ± 0.1
VO_2max_ (mL.kg^−1^.min^−1^)	44.2 ± 2.8
BF (%)	20.7 ± 4.9
BMI (kg/m^2^)	15.2 ± 1.6
HR_max_ (bpm)	202.7 ± 10.7
RPE (A.U.)	4.0 ± 0.1
s-RPE (A.U.)	301.2 ± 73.02
All-training duration (min)	7354 ± 1453
Average-training duration (min)	75.81 ± 14.98
AW (A.U.)	1285 ± 68.1

HR_max_ = Heart rate maximum; VO_2max_ = maximal oxygen consumption; BMI = body mass index; AW = accumulated acute workload in the season; BF% = body fat percentage; RPE = rate of perceived exertion; TD = training duration; A.U. = arbitrary units.

**Table 2:** Pairwise comparisons between the established subgroups (central defenders vs. central midfielder vs. striker vs. wide defender vs. wide midfielder) on the basis of age, anthropometric and maturational characteristics, and results obtained in the fitness tests performed.

Variables	CD (Mean ± SD)	CM (Mean ± SD)	WD (Mean ± SD)	WM (Mean ± SD)	ST (Mean ± SD)	p (ES)	Pairwise Comparisons (p)	ES
Age (years)	13.33 ± 0.17	13.13 ± 0.34	13.28 ± 0.15	13.26 ± 0.13	13.30 ± 0.20	0.885 (0.96)	CD vs. CM: 0.514 CD vs. WD: 0.742 CD vs. WM: 0.650 CD vs. ST: 0.971 CM vs. WD: 0.657 CM vs. WM: 0.650 CM vs. ST: 0.542 WD vs. WM: >0.999 WD vs. ST: >0.999 WM vs. ST: 0.821	CD vs. CM: 0.74 medium CD vs. WD: 0.31 small CD vs. WM: 0.17 trivial CD vs. ST: 0.16 trivial CM vs. WD: 0.57 small CM vs. WM: 0.53 small CM vs. ST: 0.58 small WD vs. WM: 0.14 trivial WD vs. ST: 0.11 trivial WM vs. ST: 0.25 small
Weight (kg)	55.75 ± 5.56	45.00 ± 7.70	51.25 ± 5.18	45.40 ± 3.84	59.67 ± 5.50	**0.032** (0.43)	CD vs. CM: 0.142 CD vs. WD: 0.342 **CD vs. WM: 0.039** CD vs. ST: 0.457 CM vs. WD: 0.342 CM vs. WM: 0.851 CM vs. ST: 0.057 WD vs. WM: 0.079 WD vs. ST: 0.228 **WM vs. ST: 0.035**	CD vs. CM: 1.60 large CD vs. WD: 0.83 medium CD vs. WM: 2.22 very large CD vs. ST: 0.70 medium CM vs. WD: 0.95 medium CM vs. WM: 0.06 trivial CM vs. ST: 2.12 very large WD vs. WM: 1.30 large WD vs. S: 1.58 large WM vs. ST: 3.19 very large
Height (cm)	175.5 ± 3.69	156.0 ± 5.59	168.5 ± 12.12	156.0 ± 6.04	178.7 ± 7.09	**0.016** (2.16)	**CD vs. CM: 0.028** CD vs. WD: 0.400 **CD vs. WM: 0.015** CD vs. ST: 0.457 CM vs. WD: 0.228 CM vs. WM: >0.999 CM vs. ST: 0.057 WD vs. WM: 0.190 WD vs. ST: 0.228 **WM vs. ST: 0.035**	CD vs. CM: 4.11 very large CD vs. WD: 0.78 medium CD vs. WM: 3.77 very large CD vs. ST: 0.60 medium CM vs. WD: 1.32 large CM vs. WM: 0.00 trivial CM vs. ST: 3.63 very large WD vs. WM: 1.36 large WD vs. ST: 0.98 moderate WM vs. ST: 3.54 very large
Seated height (cm)	91.25 ± 5.73	86.50 ± 6.45	91.00 ± 6.48	81.00 ± 2.23	92.33 ± 2.51	**0.039** (1.64)	CD vs. CM: 0.342 CD vs. WD: 0.971 **CD vs. WM: 0.023** CD vs. ST: 0.942 CM vs. WD: 0.400 **CM vs. WM: 0.031** CM vs. ST: 0.257 **WD vs. WM: 0.023** WD vs. ST: >0.999 **WM vs. ST: 0.017**	CD vs. CM: 0.77 medium CD vs. WD: 0.04 trivial CD vs. WM: 2.48 very large CD vs. ST: 0.22 small CM vs. WD: 0.69 medium CM vs. WM: 1.20 medium CM vs. ST: 1.11 medium WD vs. WM: 2.19 very large WD vs. ST: 0.25 small WM vs. ST: 4.85 very large
PHV (age)	13.45 ± 0.73	13.40 ± 0.65	13.10 ± 0.36	13.04 ± 0.39	13.42 ± 0.55	0.763 (0.81)	CD vs. CM: >0.999 CD vs. WD: 0.885 CD vs. WM: 0.603 CD vs. ST: 0.857 CM vs. WD: 0.685 CM vs. WM: 0.412 CM vs. ST: 0.857 WD vs. WM: 0.904 WD vs. ST: 0.628 WM vs. ST: 0.250	CD vs. CM: 0.07 trivial CD vs. WD: 0.60 medium CD vs. WM: 0.73 medium CD vs. ST: 0.04 trivial CM vs. WD: 0.57 small CM vs. WM: 0.69 medium CM vs. ST: 0.03 trivial WD vs. WM: 0.15 trivial WD vs. ST: 0.71 medium WM vs. ST: 0.84 medium
Maturity (years)	−0.13 ± 0.57	−0.26 ± 0.82	0.18 ± 0.31	0.20 ± 0.38	−0.13 ± 0.76	0.898 (0.98)	CD vs. CM: >0.999 CD vs. WD: 0.742 CD vs. WM: 0.730 CD vs. ST: >0.999 CM vs. WD: 0.685 CM vs. WM: 0.412 CM vs. ST: 0.857 WD vs. WM: 0.730 WD vs. ST: 0.628 WM vs. ST: 0.785	CD vs. CM: 0.18 trivial CD vs. WD: 0.67 medium CD vs. WM: 0.70 medium CD vs. ST: 0.00 trivial CM vs. WD: 0.71 medium CM vs. WM: 0.75 medium CM vs. ST: 0.16 trivial WD vs. WM: 0.05 trivial WD vs. ST: 0.57 small WM vs. ST: 0.61 medium
Weight/height ratio	31.93 ± 1.40	27.70 ± 2.55	23.60 ± 16.06	31.16 ± 3.74	30.73 ± 0.90	0.315 (0.45)	**CD vs. CM: 0.028** CD vs. WD: 0.485 CD vs. WM: >0.999 CD vs. ST: 0.400 CM vs. WD: 0.685 CM vs. WM: 0.285 CM vs. ST: 0.114 WD vs. WM: 0.904 WD vs. ST: 0.685 WM vs. ST: 0.785	CD vs. CM: 2.05 very large CD vs. WD: 0.73 medium CD vs. WM: 0.25 small CD vs. ST: 0.98 medium CM vs. WD: 0.35 small CM vs. WM: 1.05 medium CM vs. ST: 1.47 large WD vs. WM: 0.69 moderate WD vs. ST: 0.57 small WM vs. ST: 0.13 trivial
Height/weight ratio	44.65 ± 0.61	45.28 ± 1.57	34.05 ± 22.81	44.98 ± 2.96	43.20 ± 0.88	0.449 (0.29)	CD vs. CM: 0.285 CD vs. WD: 0.485 CD vs. WM: 0.674 CD vs. ST: 0.171 CM vs. WD: 0.685 CM vs. WM: >0.999 CM vs. ST: 0.114 WD vs. WM: 0.611 WD vs. ST: 0.714 WM vs. ST: 0.392	CD vs. CM: 0.52 small CD vs. WD: 0.65 medium CD vs. WM: 0.14 trivial CD vs. ST: 1.98 large CM vs. WD: 0.69 medium CM vs. WM: 0.12 trivial CM vs. ST: 1.55 large WD vs. WM: 0.72 medium WD vs. ST: 0.51 small WM vs. ST: 0.72 medium
Triceps (mm)	9.87 ± 2.39	11.38 ± 3.35	9.00 ± 2.16	13.30 ± 4.29	10.83 ± 3.25	0.688 (0.72)	CD vs. CM: >0.999 CD vs. WD: >0.999 CD vs. WM: 0.166 CD vs. ST: 0.571 CM vs. WD: 0.628 CM vs. WM: 0.976 CM vs. ST: 0.628 WD vs. WM: 0.254 WD vs. ST: 0.771 WM vs. ST: 0.392	CD vs. CM: 0.51 small CD vs. WD: 0.38 small CD vs. WM: 0.95 medium CD vs. ST: 0.34 small CM vs. WD: 0.84 medium CM vs. WM: 0.49 small CM vs. ST: 0.16 trivial WD vs. WM: 1.21 large WD vs. ST: 0.69 medium WM vs. ST: 0.62 medium
Subscapular (mm)	9.37 ± 1.25	10.25 ± 3.12	10.50 ± 3.10	14.40 ± 3.73	11.67 ± 0.57	0.178 (0.84)	CD vs. CM: 0.657 CD vs. WD: 0.371 **CD vs. WM: 0.039** CD vs. ST: 0.085 CM vs. WD: 0.914 CM vs. WM: 0.127 CM vs. ST: 0.428 WD vs. WM: 0.317 WD vs. ST: 0.971 WM vs. ST: 0.625	CD vs. CM: 0.37 small CD vs. WD: 0.47 small CD vs. WM: 1.71 large CD vs. ST: 2.22 very large CM vs. WD: 0.08 trivial CM vs. WM: 1.19 medium CM vs. ST: 0.58 small WD vs. WM: 1.12 moderate WD vs. ST: 0.48 small WM vs. ST: 0.89 medium
BF %	17.75 ± 1.15	21.57 ± 7.16	18.44 ± 2.37	24.72 ± 5.68	20.36 ± 3.79	0.385 (0.20)	CD vs. CM: >0.999 CD vs. WD: 0.371 CD vs. WM: 0.087 CD vs. ST: 0.628 CM vs. WD: 0.885 CM vs. WM: 0.682 CM vs. ST: 0.914 WD vs. WM: 0.095 WD vs. ST: 0.400 WM vs. ST: 0.250	CD vs. CM: 0.74 medium CD vs. WD: 0.37 small CD vs. WM: 1.59 large CD vs. ST: 1.02 medium CM vs. WD: 0.58 small CM vs. WM: 0.49 small CM vs. ST: 0.20 small WD vs. WM: 1.37 large WD vs. ST: 0.63 medium WM vs. ST: 0.85 medium
AW-first half (A.U.)	1335 ± 57.39	1411 ± 128.0	1335 ± 59.95	1434 ± 69.45	1404 ± 8.44	0.210 (0.75)	CD vs. CM: 0.485 CD vs. WD: 0.885 CD vs. WM: 0.063 CD vs. ST: 0.400 CM vs. WD: 0.485 CM vs. WM: 0.904 CM vs. ST: >0.999 WD vs. WM: 0.111 WD vs. ST: 0.057 WM vs. ST: 0.392	CD vs. CM: 0.76 medium CD vs. WD: 0.00 trivial CD vs. WM: 1.53 trivial CD vs. ST: 1.54 large CM vs. WD: 0.76 medium CM vs. WM: 0.23 trivial CM vs. ST: 0.07 trivial WD vs. WM: 1.51 large WD vs. ST: 1.47 large WM vs. ST: 0.52 small
AW-second half (A.U.)	1168 ± 58.49	1169 ± 101.9	1214 ± 62.22	1176 ± 66.71	1208 ± 61.64	0.904 (0.99)	CD vs. CM: 0.885 CD vs. WD: 0.685 CD vs. WM: 0.555 CD vs. ST: 0.400 CM vs. WD: 0.885 CM vs. WM: >0.999 CM vs. ST: 0.857 WD vs. WM: 0.555 WD vs. ST: >0.999 WM vs. ST: >0.999	CD vs. CM: 0.01 trivial CD vs. WD: 0.76 medium CD vs. WM: 0.12 trivial CD vs. ST: 0.66 medium CM vs. WD: 0.53 small CM vs. WM: 0.084 trivial CM vs. ST: 0.44 trivial WD vs. WM: 0.58 small WD vs. ST: 0.09 medium WM vs. ST: 0.49 small
10 m (s)	1.16 ± 0.04	1.35 ± 0.07	1.26 ± 0.01	1.35 ± 0.08	1.29 ± 0.07	**0.025** (1.90)	**CD vs. CM: 0.028** **CD vs. WD: 0.028** **CD vs. WM: 0.031** CD vs. ST: 0.085 CM vs. WD: 0.057 CM vs. WM: 0.904 CM vs. ST: 0.628 WD vs. WM: 0.190 WD vs. ST: 0.628 WM vs. ST: 0.464	CD vs. CM: 3.33 very large CD vs. WD: 3.43 very large CD vs. WM: 2.88 very large CD vs. ST: 2.40 very large CM vs. WD: 1.80 large CM vs. WM: 0.00 trivial CM vs. ST: 0.85 medium WD vs. WM: 1.48 large WD vs. ST: 0.66 medium WM vs. ST: 0.78 medium
30 m (s)	3.48 ± 0.03	3.85 ± 0.16	3.58 ± 0.07	3.85 ± 0.19	3.60 ± 0.17	**0.017** (2.15)	**CD vs. CM: 0.028** CD vs. WD: 0.057 **CD vs. WM: 0.02** CD vs. ST: 0.285 CM vs. WD: 0.057 CM vs. WM: 0.904 CM vs. ST: 0.114 WD vs. WM: 0.119 WD vs. ST: >0.999 WM vs. ST: 0.142	CD vs. CM: 3.21 very large CD vs. WD: 1.85 large CD vs. WM: 2.55 very large CD vs. ST: 1.09 medium CM vs. WD: 2.18 very large CM vs. WM: 0.00 trivial CM vs. ST: 1.52 large WD vs. WM: 1.79 large WD vs. ST: 0.16 trivial WM vs. ST: 1.36 large
V IFT (km/h)	17.00 ± 1.58	16.00 ± 0.91	17.00 ± 1.58	16.10 ± 1.81	16.00 ± 1.73	0.760 (0.81)	CD vs. CM: 0.457 CD vs. WD: >0.999 CD vs. WM: 0.539 CD vs. ST: 0.571 CM vs. WD: 0.457 CM vs. WM: 0.754 CM vs. ST: 0.914 WD vs. WM: 0.539 WD vs. ST: 0.571 WM vs. ST: >0.999	CD vs. CM: 0.77 medium CD vs. WD: 0.00 trivial CD vs. WM: 0.52 small CD vs. ST: 0.60 medium CM vs. WD: 0.77 medium CM vs. WM: 0.06 trivial CM vs. ST: 0.00 trivial WD vs. WM: 0.52 small WD vs. ST: 0.60 medium WM vs. ST: 0.05 trivial
VO_2max_ (mL.kg^−1^ min^−1^)	45.25 ± 2.98	43.08 ± 1.56	45.40 ± 2.90	43.96 ± 3.47	43.27 ± 3.40	0.700 (0.74)	CD vs. CM: 0.485 CD vs. WD: >0.999 CD vs. WM: 0.730 CD vs. ST: 0.457 CM vs. WD: 0.485 CM vs. WM: 0.730 CM vs. ST: 0.628 WD vs. WM: 0.730 WD vs. ST: 0.228 WM vs. ST: 0.785	CD vs. CM: 0.91 medium CD vs. WD: 0.05 trivial CD vs. WM: 0.39 small CD vs. ST: 0.62 medium CM vs. WD: 0.99 medium CM vs. WM: 0.31 small CM vs. ST: 0.07 trivial WD vs. WM: 0.44 small WD vs. ST: 0.68 medium WM vs. ST: 0.20 small
HR_max_ (bpm)	206.0 ± 5.77	205.0 ± 8.40	200.8 ± 11.79	199.6 ± 17.95	202.7 ± 4.72	0.959 (1.07)	CD vs. CM: >0.999 CD vs. WD: 0.828 CD vs. WM: 0.904 CD vs. ST: 0.714 CM vs. WD: 0.828 CM vs. WM: >0.999 CM vs. ST: 0.714 WD vs. WM: 0.507 WD vs. ST: 0.685 WM vs. ST: 0.785	CD vs. CM: 0.13 trivial CD vs. WD: 0.56 small CD vs. WM: 0.45 small CD vs. ST: 0.61 medium CM vs. WD: 0.41 small CM vs. WM: 0.36 small CM vs. ST: 0.32 small WD vs. WM: 0.07 trivial WD vs. ST: 0.19 trivial WM vs. ST: 0.20 small

BF% = body fat; 10 m = 10 meters; med = median; 30 m = 30 meters; HR_max_ = heart rate maximum; PHV = peak height velocity; V IFT = the final velocity of intermittent fitness test 30–15; A.U. = arbitrary units; CD = central defender; CM = central midfielder; ST = striker; WD = wide defender; WM = wide midfielder. Significant differences (*p* ≤ 0.05) are highlighted in bold.

**Table 3:** Matrix of correlations of variables’ accumulated workload (AW) based on periods (i.e., first and second halves) with maturity (i.e., maturity offset and peak height velocity; PHV), linear sprints (i.e., 10 m and 30 m), maximum oxygen uptake (VO_2max_), maximum heart rate (HR_max_), body fat, RPE, s-RPE, TD-top, and TD-average.

Variables	Maturity	PHV	AW1	AW2	10 m	30 m	VO_2max_	HR_max_	BF%	RPE	TD-Top	TD-Mean	s-RPE
maturity	1.000												
PHV	−0.190	1.000											
AW1	−0.159	0.361	1.000										
AW2	−0.215	0.290	**0.551**	1.000									
10m	−0.337	0.351	0.084	−0.104	1.000								
30m	−0.031	0.231	0.173	−0.121	**0.769**	1.000							
VO_2max_	0.266	−0.210	**−0.480**	−0.419	−0.081	−0.054	1.000						
HR_max_	0.112	−0.110	−0.389	−0.236	0.075	−0.021	**0.495**	1.000					
BF%	−0.302	0.271	0.344	0.056	0.399	0.241	−0.230	**−0.469**	1.000				
RPE	0.431	−0.270	0.059	−0.072	−0.109	0.055	0.238	−0.259	0.417	1.000			
TD-top	−0.072	0.030	−0.066	−0.194	−0.098	−0.092	0.429	−0.204	0.154	0.294	1.000		
TD-mean	−0.072	0.030	−0.066	−0.194	−0.098	−0.092	0.429	−0.204	0.154	0.294	**1.000**	1.000	
s-RPE	0.190	−0.090	0.065	−0.084	−0.183	−0.087	0.375	−0.264	0.401	**0.853**	**0.666**	**0.666**	1.000

Significant differences (*p* ≤ 0.05) are highlighted in bold. PHV = peak height velocity; AW = acute workload; 1 = first half of season and 2 = second half of season, respectively.

## Data Availability

The data presented in this study are available on reasonable request from the corresponding author. The data are not publicly available due to privacy reasons.
